# Why precision oncology works: lessons from gastrointestinal stromal tumours

**DOI:** 10.37349/etat.2026.1002378

**Published:** 2026-07-06

**Authors:** Si Ying Adelina Ho, Alwyn Hong Sheng Tok, Hui Qi Megan Kimberly Han, Jens Samol, Vishal G Shelat

**Affiliations:** IRCCS Azienda Ospedaliero-Universitaria di Bologna, Italy; ^1^Ministry of Health Holdings, Singapore 139691, Singapore; ^2^Yong Loo Lin School of Medicine, National University of Singapore, Singapore 119228, Singapore; ^3^Department of Medical Oncology, Tan Tock Seng Hospital, Singapore 308433, Singapore; ^4^Lee Kong Chian School of Medicine, Nanyang Technological University, Singapore 308232, Singapore; ^5^Johns Hopkins University, Baltimore, MD 21287, USA; ^6^Department of General Surgery, Tan Tock Seng Hospital, Singapore 308433, Singapore

**Keywords:** GIST, molecular classification, *KIT* mutation, *PDGFRA* mutation, tyrosine kinase inhibitors, targeted therapy, treatment sequencing

## Abstract

Gastrointestinal stromal tumours (GISTs) are the clearest solid-tumour model of precision oncology because diagnosis, prognosis, and treatment are strongly shaped by molecular genotype. The discovery of activating mutations in *KIT* proto-oncogene receptor tyrosine kinase (*KIT*) and platelet-derived growth factor receptor alpha (*PDGFRA*) transformed management by enabling genotype-directed use of tyrosine kinase inhibitors (TKIs) across localized and advanced disease. This review summarizes how molecular classification informs contemporary GIST care, from diagnostic work-up and risk stratification to neoadjuvant, adjuvant, and metastatic treatment planning. *KIT* exon 11 mutations generally predict sensitivity to standard-dose imatinib, whereas *KIT* exon 9 tumours may benefit from dose escalation. *PDGFRA* D842V confers primary resistance to imatinib but sensitivity to avapritinib, illustrating the clinical value of mutation-specific therapy. We also review *KIT*/*PDGFRA*-wild-type GISTs, including succinate dehydrogenase (*SDH*)-deficient, neurofibromin 1 (*NF1*)-associated, B-Raf proto-oncogene (*BRAF*)-mutant, and neurotrophic tyrosine receptor kinase (*NTRK*)-rearranged subtypes, where extended molecular testing is increasingly important. Surgery remains central in localized disease, but operative timing, extent of resection, and use of neoadjuvant therapy should be individualized according to tumour site, rupture risk, technical feasibility, and genotype. In advanced disease, sequential use of imatinib, sunitinib, regorafenib, ripretinib, and selected mutation-specific agents reflects evolving resistance biology and the need for ongoing molecular interpretation. Emerging tools such as broader genomic profiling and liquid biopsy may further refine treatment selection. GIST therefore demonstrates that precision oncology is most effective when molecular diagnostics, surgery, systemic therapy, and multidisciplinary decision-making are integrated across the full disease course.

## Introduction

Gastrointestinal stromal tumours (GISTs) represent the most common subtype of mesenchymal neoplasms arising within the gastrointestinal (GI) tract and are believed to originate from the interstitial cells of Cajal, most notably in the stomach [1, 2]. With an estimated incidence of up to 20 cases per million population and accounting for approximately 5% of all sarcomas, GISTs predominantly affect older adults, with a slight male predominance [2].

The clinical presentation of GISTs is heterogeneous and may include GI bleeding secondary to mucosal ulceration, local symptoms such as early satiety and nausea, constitutional symptoms, and incidental detection during imaging or endoscopy. Since GISTs often present asymptomatically, their true incidence may be underestimated. This is evident from autopsy studies suggesting that the prevalence of subclinical GISTs can reach up to 22.5% in individuals over 50 years of age [3]. Given the wide spectrum of clinical presentations and frequent occurrence of incidental findings, robust risk stratification is essential to guide management strategies and prognostication. GIST has become the prototypical model of precision oncology in solid tumours as clinical outcomes are driven primarily by oncogenic kinase genotype and resistance biology. In this review, we demonstrate how molecular classification can guide the management of GISTs through predicting treatment efficacy, dose selection, and adaptive sequencing as resistance evolves, while also highlighting factors that influence real world implementation.

While most GISTs are sporadic, a subset can occur in hereditary syndromes, such as the Carney triad and Carney-Stratakis syndrome [4]. Mutation-based testing in such hereditary syndromes ensures preliminary detection of molecular alterations, which is especially critical in younger patients. This is because rare familial GIST syndromes due to germline *KIT* proto-oncogene receptor tyrosine kinase (*KIT*) or platelet-derived growth factor receptor alpha (*PDGFRA*) mutations are characterised by early onset, multi-focal tumours, and distinctive dermatological manifestations. Hence, early recognition of these entities can better guide the initiation of further genetic counselling, chronic surveillance, and family screening.

Adolescent and young adult (AYA) GIST warrants explicit consideration because its biology is more frequently enriched for kinase-wild-type and syndromic subtypes [e.g., succinate dehydrogenase (*SDH*)-deficient disease], where standard *KIT*-centric assumptions may underperform [5]. In such presentations, genetic counselling is an integral component of precision care, informing surveillance strategies and family risk assessment in addition to systemic therapy selection.

Current therapeutic decision-making is often guided by GIST risk factors (Fletcher et al. [6], modified by Joensuu [7]). According to the National Institute of Health (NIH) consensus led by Fletcher et al. [6], tumour size and mitotic count remain the most widely-accepted morphologic predictors of recurrence risk. However, very small tumours with low mitotic rates may rarely metastasise, highlighting that these factors are better applied for risk stratification rather than labeling any GIST as definitively benign. Additional GIST risk factors include tumours of more than 5 cm, the extra-gastric region as a site of origin, tumour rupture or perforation, and mitotic rate of more than 5 per 50 high-power fields. Joensuu [7] further emphasised the importance of tumour site and rupture, noting that gastric GISTs recur less frequently than extra-gastric tumours of comparable size and mitotic count, while tumour rupture confers a substantially increased risk of recurrence. In addition to clinico-pathologic risk stratification, molecular profiling has increasingly emerged as an important tool that can refine prognostication and guide therapeutic decision making, including dose optimisation and treatment sequencing.

## Molecular classification and testing

The most clinically significant molecular alterations in GISTs involve activating mutations in the *KIT* proto-oncogene (CD117) and *PDGFRA*, which are expressed in more than 75% and 8% [1] of all cases, respectively. Importantly, each molecular alteration responds distinctly to different dosing and types of tyrosine kinase inhibitor (TKI), with distinct resistance patterns. The College of American Pathologists guidelines recommend *KIT* and *PDGFRA* mutational testing in all GIST patients with high-risk features or metastatic disease to guide TKI selection [8]. This is because immunohistochemical expression of *KIT* (CD117) is no longer sufficient to predict treatment response and must not replace molecular profiling. In cases of *KIT*/*PDGFRA*-wild-type GISTs, *SDH* subunit B (*SDHB*) immunohistochemistry (IHC) should be routinely performed to detect *SDH* deficiency, followed by sequencing or methylation testing as appropriate. Triple-negative cases should be evaluated for rare drivers such as B-Raf proto-oncogene (*BRAF*), serine/threonine kinase, neurofibromin 1 (*NF1*), or neurotrophic tyrosine receptor kinase (*NTRK*) fusions. Although uncommon, *NTRK*-rearranged GISTs are clinically significant, particularly in paediatric and *SDH*-wild-type GISTs, and respond to tropomyosin receptor kinase (TRK) inhibitors (subset of TKIs) such as larotrectinib or entrectinib. Similarly, *BRAF*-mutated GISTs may benefit from *BRAF* or mitogen-activated protein kinase kinase (MEK) inhibitors, while emerging fibroblast growth factor receptor 1 (FGFR1)-altered GISTs represent another targetable subgroup under active investigation [9]. In practice, mutational testing is further prioritised based on its ability to influence treatment decisions, such as TKI choice or dose, perioperative strategy, and sequencing at progression, with reflex workflows implemented for *KIT*/*PDGFRA*-wild-type disease.

*KIT* mutations most notably occur in exons 11, 9, 13, and 17. Mutations in exon 11, which encodes the juxtamembrane domain, account for approximately 60–70% of GISTs. These mutations commonly involve deletions at codons 557–558 (Trp557_Lys558del) and are particularly prevalent for GISTs in the gastric region [8]. Exon 9 mutations represent 10–20% of cases and frequently involve duplications at codons 502–503. They predominantly involve small intestinal GISTs and commonly require higher-dose imatinib (800 mg/day). MetaGIST meta analysis of 1,640 patients with advanced GIST found that imatinib 800 mg daily (versus 400 mg daily) delivered a small but significant progression-free survival (PFS) advantage with no overall survival (OS) benefit, with the benefit limited to *KIT* exon 9 mutant disease, supporting pre-treatment mutational testing to help individualise starting dose [10]. Mutations in *KIT* exons 13 and 17 are less common and are frequently identified as secondary resistance mutations arising during imatinib treatment.

*PDGFRA* mutations occur in approximately 5–10% of GISTs and are most commonly observed in gastric tumours with epithelioid or mixed histological features. The most prevalent *PDGFRA* mutation is the exon 18 p.D842V substitution, which confers primary resistance to imatinib and most other TKIs, with the notable exception of avapritinib. In contrast, exon 12 *PDGFRA* mutations (approximately 1–2% of cases), are generally sensitive to imatinib, while exon 14 mutations are exceedingly rare (< 0.1%).

In addition to *KIT* and *PDGFRA*, several immunohistochemical markers play a supportive diagnostic role. Discovered On GIST-1 (DOG1), which had a specificity of 100%, was found to be positive in 96% of GISTs, while protein kinase C theta (PKC-theta), with a specificity of 80%, was positive in 90.5% of cases [11]. *SDH*-deficient GISTs, which are typically gastric in nature and more commonly affect children or young adults, often demonstrate resistance to imatinib due to absence of *KIT*/*PDGFRA* mutations and are characterised by overexpression of insulin-like growth factor 1 receptor (IGF1R) [12].

**Figure 1:**
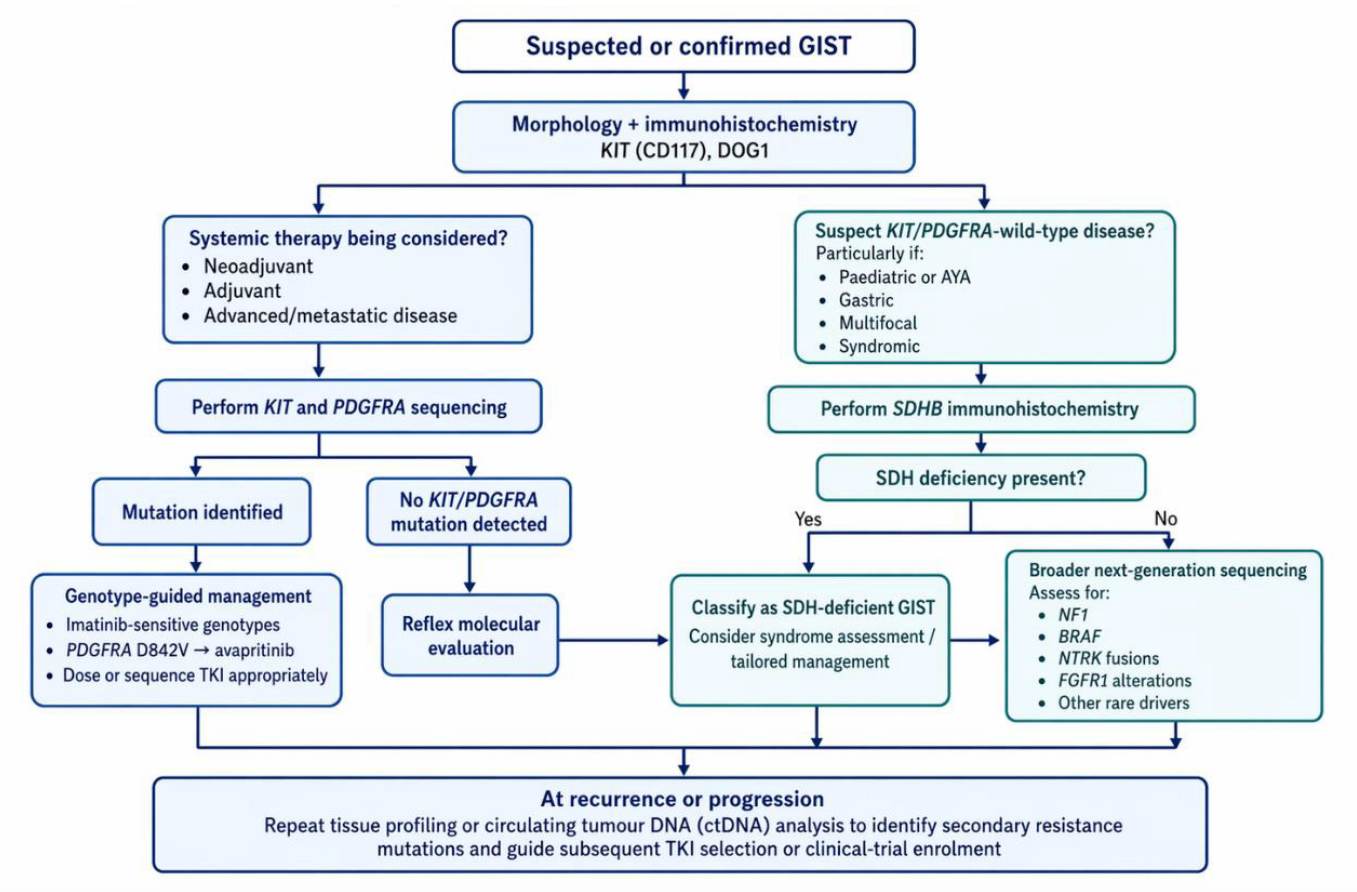
**Molecular testing workflow for suspected or confirmed GIST.** AYA: adolescent and young adult; DOG1: Discovered On GIST-1; FGFR1: fibroblast growth factor receptor 1; GIST: gastrointestinal stromal tumour; *KIT*: *KIT* proto-oncogene receptor tyrosine kinase; *NF1*: neurofibromin 1; *NTRK*: neurotrophic tyrosine receptor kinase; *PDGFRA*: platelet-derived growth factor receptor alpha; *SDH*: succinate dehydrogenase; *SDHB*: succinate dehydrogenase subunit B; TKI: tyrosine kinase inhibitor.

Advances in mutational profiling have expanded the spectrum of actionable targets in GISTs beyond *KIT* and *PDGFRA*. The incorporation of next-generation sequencing into routine diagnostic workflows enables the identification of less common yet clinically relevant alterations. These include *BRAF* mutations, *NTRK* gene fusions, *NF1* mutations, and FGFR1 rearrangements. Each of these molecular aberrations carries distinct therapeutic implications, highlighting the growing importance of precision oncology in the management of GISTs. Figure 1 summarises a practical molecular testing workflow for suspected or confirmed GIST, integrating diagnostic IHC, *KIT*/*PDGFRA* sequencing, *SDHB* IHC, reflex next-generation sequencing, and repeat profiling at recurrence or progression.

## Surgical management options for GISTs

While molecular profiling informs systemic therapy selection, complete surgical resection is the standard treatment for resectable localized GIST, serving either a curative or palliative role depending on disease extent and symptom burden. In localised, non-high risk GISTs, complete [microscopically margin-negative resection (R0)] resection is associated with excellent outcomes, with reported five-year recurrence-free survival (RFS) rates of up to 99.1% [13]. Given the unfavourable prognostic outcomes associated with metastatic disease, timely and complete excision of localised GISTs remains crucial in optimising long-term outcomes.

Although there is broad consensus that GISTs greater than 2 cm should be surgically resected whenever feasible, the optimal management of small GISTs, particularly those measuring less than 2 cm, remains controversial. Guidelines converge on resection for higher-risk phenotypes but diverge for incidentally detected small gastric lesions. Both the Asian Consensus and the European Society for Medical Oncology (ESMO) guidelines endorse surgical resection as the primary treatment modality for tumours exceeding 2 cm, those that are growing or display malignant features, or those arising from extra-gastric sites [14, 15]. For incidentally detected gastric GISTs measuring less than 2 cm without malignant features, the Asian Consensus guidelines support active surveillance rather than immediate surgical intervention [14]. On the other hand, ESMO underscores the inherent malignant potential of all GISTs and advocates for an individualised management approach for tumours under 2 cm, taking into account patient factors, tumour characteristics, and local expertise [15]. Published estimates of GIST tumour doubling time are limited and variable; the best-cited series reported a median doubling time of 17.2 months overall, with much faster growth in high-risk tumours (3.9 months) and slower growth in low-risk tumours (24.0 months) [16]. Given the variable growth kinetics of small GISTs and the low-risk profile of many incidentally detected gastric lesions measuring less than 2 cm, short-interval surveillance with shared decision-making is a reasonable strategy in carefully selected patients. This approach should integrate tumour size, location, interval growth, endoscopic ultrasonographic (EUS) features, patient comorbidity, and local expertise, recognising that not all small lesions require immediate intervention. This conservative approach is also consistent with National Comprehensive Cancer Network (NCCN) guidance, which supports observation for most very small gastric GISTs measuring less than 2 cm when they are asymptomatic and lack high-risk EUS features, while reserving resection for symptomatic lesions, tumours concerning EUS findings, interval growth, or non-gastric location [17]. Increasingly, molecular subtype may also influence surgical strategy. Tumours harbouring *KIT* exon 11 mutations tend to respond well to neoadjuvant imatinib, which may permit organ-preserving surgery in anatomically challenging locations such as the gastro-oesophageal junction or rectum. Conversely, tumours with primary imatinib resistance (e.g., *PDGFRA* D842V) may derive less benefit from preoperative TKI therapy and are therefore more likely to require upfront definitive resection when technically feasible.

For incidentally detected small gastric subepithelial lesions, EUS can refine decision-making by confirming origin from the muscularis propria, more accurately defining tumour size, and identifying concerning features such as irregular margins, ulceration, bleeding, cystic change, necrosis, or heterogeneous echogenicity [14]. In addition, when tissue diagnosis is required before neoadjuvant therapy, EUS-guided biopsy is generally preferred to percutaneous biopsy because it can provide histology while minimising the risk of tumour spillage. One possible option for smaller tumours is endoscopic resection, although its use remains controversial because of the risks of incomplete excision, tumour rupture or spillage, bleeding, and perforation. Accordingly, the decision to pursue endoscopic resection should be individualised based on tumour characteristics, technical feasibility, operator experience, and patient preference. Nevertheless, reports of success in highly selected small lesions exist, and with advances in endoscopic techniques and operator expertise, endoscopic resection may be considered a therapeutic option for selected small GISTs requiring treatment. A recent 2024 study involving 233 patients reported successful and safe R0 resection in 93% of cases, although the cohort was limited to tumours smaller than 2 cm and confined to the muscularis propria [18]. For selected small gastric GISTs, a meta-analysis of 11 studies reported that endoscopic resection for tumours under 2 cm achieved pooled complete resection, post-operative adverse event, and recurrence rates of 97%, 8%, and 3% respectively, with shorter operative time than laparoscopic resection and no significant differences in blood loss, length of stay, post-operative complications, or recurrence in comparative studies [19]. Thus, both endoscopic and surgical options are reasonable depending on local resources, expertise, and experience. Within surgical practice, the choice of access should remain subordinate to the principles of safety, oncologic adequacy, and technical soundness. Laparoscopic and robotic approaches are enabling modalities rather than markers of superiority. Where an open operation offers the most reliable route to complete and safe resection, it should be embraced as an appropriate strategy rather than perceived as a lesser alternative.

R0 resection is recommended in surgical oncology management plans, including for GIST. However, clinical decisions are made considering technical, oncological, and functional aspects. For example, a duodenal GIST may be managed with a wedge resection rather than pancreaticoduodenectomy, which is more morbid procedure. Sometimes, despite best attempts to avoid, tumour comes close to resected margins [microscopically margin-positive resection (R1) resection]. While this is not optimal situation, re-resection is not routinely recommended solely for microscopic positivity and decisions should be individualized as evidence suggests that microscopic margin positivity may not always translate to worse outcomes. In a retrospective cohort study of 48 gastric GISTs, laparoscopic resection was associated with higher R1 resection rates than open surgery, yet no recurrence or metastases were observed during follow-up, and OS was not compromised [20]. Nevertheless, metastatic disease remains a major determinant of prognosis. In a Surveillance, Epidemiology, and End Results (SEER) based analysis of 4,224 GIST patients diagnosed between 2010 and 2015, the median OS for those with liver, bone, and lung metastases was 49, 18, and 20 months, respectively, underscoring the adverse prognostic impact of metastatic spread [21]. Therefore, the decision to pursue re-resection versus initiation of adjuvant therapy must be carefully evaluated and weighed on a case-by-case basis.

## Advanced disease: systemic therapy, sequencing, and resistance

In addition to localised disease, the management of GIST must also consider advanced presentations, including unresectable or metastatic tumours. The B2222 [22] and S0033 [23] trials established imatinib as an effective therapy for advanced GISTs, inaugurating genotype-directed TKI treatment in solid tumours. In the B2222 study, 147 patients with unresectable or metastatic GIST received imatinib at doses of 400 mg or 600 mg daily. It achieved a 53.7% partial response rate and 27.9% stable disease rate, with early resistance at 13.6% and an estimated 1 year survival of 88%, demonstrating substantial clinical benefit with imatinib therapy. No significant differences in efficacy were observed between the two dosing regimens [22]. Similarly, the phase III S0033 trial randomised 694 patients with unresectable or metastatic GIST to receive imatinib 400 mg once daily or 400 mg twice daily, with a median follow-up of 4.5 years. Although the higher dose regimen led to more grade 3 to 5 toxicities, dose escalation after progression of GIST achieved response or stable disease in approximately one third of the patients, reinforcing the efficacy of imatinib for advanced GISTs [23]. With such evidence, the following sections discuss the role of imatinib in the management of advanced GISTs, as well as the sequencing of other TKIs in cases of imatinib resistance.

## Adjuvant imatinib after complete resections

With respect to adjuvant imatinib, its efficacy in reducing GIST recurrence and improving OS following complete resection was evaluated in three phase III randomised controlled trials, namely the American College of Surgeons Oncology Group (ACOSOG) Z9001 [24], SSGX-VIII/AIO [25], European Organisation for Research and Treatment of Cancer (EORTC) 62024 [26] studies. First, the ACOSOG Z9001 study showed improved RFS of patients with entirely resected, *KIT*-positive GISTs measuring more than or equal to 3 cm, after taking 1 year of adjuvant imatinib 400 mg daily [24]. Supplementing this finding, the SSGX-VIII/AIO study prolonged the duration of treatment and constricted patient selection, comparing 1 year versus 3 years of adjuvant imatinib 400 mg daily in patients with high-risk, completely resected, *KIT*-positive GIST. The study demonstrated that three years of therapy resulted in significantly improved RFS and OS compared to only one year of therapy [25]. Sustained follow-up confirmed superior 10-year OS in the 3-year treatment group. The EORTC 62024 study reconciled the former two studies, randomising patients with intermediate or high-risk GIST to 2 years of imatinib 400 mg daily versus observation after complete resection [26]. While the study demonstrated a statistically significant improvement in RFS, no significant benefit was observed in OS or imatinib failure-free survival in this unselected intermediate- and high-risk population. Collectively, these trials support the current standard recommendation of adjuvant imatinib at 400 mg daily for three years in patients with high-risk, *KIT*-positive GISTs. Postoperative recurrence estimation can be supported by validated prognostic tools such as the Memorial Sloan Kettering Cancer Center GIST nomogram, which estimates RFS after complete resection of localised primary GIST using clinicopathological variables [27]. These tools should complement, rather than replace, Fletcher/Joensuu risk stratification, genotype, patient factors, and multidisciplinary judgement.

Neoadjuvant imatinib, as evaluated in Radiation Therapy Oncology Group (RTOG) 0132/ACRIN (American College of Radiology Imaging Network) 6665 [28], can be advantageous in both primary and metastatic/recurrent GISTs, particularly for tumours in precarious locations (e.g., gastro-oesophageal junction, duodenum, or rectum) or for larger tumours with an increased risk of rupture. It demonstrated the ability to preserve organ function and reduce post-operative morbidity. Outcomes were favourable, with high rates of disease stabilisation in both primary (83%) and metastatic (91%) groups, minimal surgical complications, and two-year PFS of 83% (primary) and 77% (metastatic).

## TKI strategies for subsequent GIST progression

Although proven to be successful in specific cases, neoadjuvant imatinib may not yield equal results when there is resistance or intolerance to it as primary treatment. Hence, secondary and tertiary agents have been explored to maintain disease control and maximise outcomes. Secondary resistance most commonly originates from accessory mutations in the adenosine triphosphate (ATP)-binding pocket (exons 13 and 14) or activation loop (exons 17 and 18) of *KIT*, necessitating the use of TKIs with broader inhibitory profiles, such as ripretinib.

One example of secondary treatment includes sunitinib. A phase III randomised controlled trial demonstrated that sunitinib slowed down tumour progression compared to placebo, with a median progression time of 27.3 weeks and 6.4 weeks, respectively [29]. However, patients on sunitinib may still experience tumour progression, hence entailing further lines of treatment. Further intolerance of imatinib and sunitinib warrants tertiary treatment using regorafenib. This is corroborated by the GRID [30] trial, which showed that regorafenib significantly improved median PFS (4.8 months versus 0.9 months in placebo) in patients with metastatic or unresectable GIST despite being on imatinib and sunitinib. Although objective response rates were modest (4.5%), the clinical benefit rate approached 50%, highlighting its role in disease control beyond radiographic response. As patients exhaust traditional TKI options, ongoing clinical trials continue to explore investigational agents and novel TKIs, expanding the therapeutic armamentarium beyond regorafenib.

Novel therapeutic strategies in GIST can be viewed in four overlapping categories. First, next-generation TKIs aim to overcome heterogeneous secondary *KIT* resistance, as illustrated by ripretinib, which has broader activity across activation-loop and ATP-binding-pocket resistance mutations. Second, mutation-specific inhibition has become clinically important, most clearly with avapritinib for *PDGFRA* D842V-mutant GIST, a subtype historically resistant to imatinib. Third, rational combination strategies are being explored to deepen response or delay resistance, including *KIT* plus MEK inhibition based on the interaction between *KIT*-MAPK signalling and Ets variant transcription factor-1 (ETV1)-dependent GIST lineage survival. Fourth, rare molecular subtypes increasingly require pathway-directed or trial-based approaches, including TRK inhibitors for *NTRK*-rearranged GIST, *BRAF*/MEK-directed treatment for *BRAF*-mutant disease, and investigational approaches for *SDH*-deficient, *NF1*-associated, FGFR-altered, or other *KIT*/*PDGFRA*-wild-type tumours. These approaches remain unevenly mature: some are already clinically established, whereas others remain early-phase or hypothesis-generating and should be considered in molecular tumour boards or clinical trials where available.

Dual-pathway inhibition has also emerged as a rational strategy in GIST. Beyond sequential single-agent TKI therapy, early studies have explored combining *KIT* inhibition with MEK inhibition to suppress downstream MAPK signalling and destabilise the lineage survival factor ETV1. ETV1 is a lineage-specific transcription factor essential for interstitial cells of Cajal development, acts as a master regulator of GIST biology, and is stabilized by *KIT*-MAPK signaling, providing the biological rationale for combined *KIT* and MEK inhibition strategies. In a phase II study of first-line imatinib plus binimetinib in treatment-naive advanced GIST, the combination achieved a Response Evaluation Criteria in Solid Tumors (RECIST) objective response rate of 69.0% (29/42), a Choi partial response rate of 95.1% (39/41), and a median PFS of 29.9 months, with manageable toxicity [31]. Notably, five of eight patients with locally advanced disease who subsequently underwent surgery achieved significant pathologic response of at least 90% treatment effect, suggesting that dual-agent therapy may deepen response beyond conventional radiographic shrinkage alone. The principal grade 3–4 toxicities included asymptomatic creatine phosphokinase elevation (79.1%), hypophosphataemia (14.0%), neutrophil decrease (9.3%), maculopapular rash (7.0%), and anaemia (7.0%).

Other feasible TKIs include ripretinib (INVICTUS [32] trial), avapritinib (NAVIGATOR [33] trial), cabozantinib (CABOGIST [34] trial), and pazopanib (PAZOGIST [35] trial). In the phase 3 INVICTUS trial (*n* = 129), ripretinib 150 mg once daily improved median PFS to 6.3 months versus 1.0 month with placebo [hazard ratio (HR) 0.15, *p* < 0.0001], produced a confirmed objective response rate of 9.4% versus 0%, and increased median OS to 15.1 months versus 6.6 months (HR 0.36) [32]. Similarly, the NAVIGATOR phase 1 trial evaluated avapritinib in patients with unresectable or metastatic *PDGFRA* D842V mutant GIST. Among 56 patients, avapritinib produced an overall response rate of 91% (51/56) and a clinical benefit rate of 98% (55/56), with median duration of response 27.6 months and median PFS 34.0 months [33]. Additional TKIs have also shown activity in later-line settings. In the phase II EORTC 1317 CaboGIST study of metastatic GIST after imatinib and sunitinib, cabozantinib met its primary endpoint with 58.5% progression free at 12 weeks and achieved a 14% partial response rate with 82% disease control and a median PFS of 5.5 months [34]. Likewise, in the randomised phase 2 PAZOGIST trial (*n* = 81) of advanced GIST resistant to imatinib and sunitinib, pazopanib 800 mg daily plus best supportive care improved investigator assessed PFS versus best supportive care alone, with 4 month PFS 45.2% versus 17.6% (HR 0.59, *p* = 0.029) and median PFS 3.4 versus 2.3 months (HR 0.59, *p* = 0.03) [35].

Although these TKIs have demonstrated meaningful clinical activity in patients with secondary resistance to standard therapy, their optimal selection still increasingly depends on the underlying resistance mutation profile. Ripretinib, a switch-control TKI, demonstrates pan-*KIT* inhibitory activity across a spectrum of secondary resistance mutations in exons 13, 14, 17, and 18. On the other hand, avapritinib is a Type I inhibitor that binds the active kinase conformation and is highly selective for *PDGFRA* D842V mutations, with limited efficacy against other *KIT* mutations. These examples highlight the distinct mechanistic differences between each TKI. Precise mutation profiling of patients can therefore guide desirable selection and sequencing of therapy, optimising efficacy and improving treatment outcomes.

Besides the sequencing of TKI therapy, alternative strategies may also be considered after disease progression. One such approach is imatinib re-challenge. This was highlighted in the RIGHT study, where imatinib re-challenge demonstrated improved PFS compared to placebo in patients who had previously progressed on therapy [36]. This observation likely reflects the continued suppression of certain *KIT*-mutated subclones within a heterogeneous tumour population. Although Nilotinib is not universally adopted, it has shown fair disease control in patients with imatinib- and sunitinib-resistant GIST in the ENESTg1 trial, and may offer benefit in select cases, particularly where access to newer agents is limited [37]. Recent emerging therapies tailored to specific molecular markers and pathways may also prove to be a viable alternative to TKI sequencing. Notably, pre-clinical studies on anti-DOG1 monoclonal antibodies have demonstrated the ability to induce apoptosis and inhibit cell migration and invasiveness in xenograft models of DOG1-expressing alimentary tract tumours [38]. Other than systemic therapies and molecular targets, locoregional modalities such as radiotherapy and chemotherapy have also been investigated, particularly for metastatic or refractory disease. Table 1 provides a summary of mutation-driven treatment pathways in GISTs, including emergent therapies targeting FGFR fusions.

**Table 1:** Mutation-driven treatment pathways in GIST.

**Gene/Mutation**	**Frequency**	**Site/Histology**	**Sensitivity to TKIs**	**Recommended Therapy**	**Resistance mechanism/testing workflow**
*KIT* exon 11	~60–70%	Stomach; spindle cell	Sensitive to imatinib 400 mg	Imatinib 400 mg daily	N/A
*KIT* exon 9	~10–20%	Small intestine; aggressive	Partially resistant to imatinib 400 mg; better with 800 mg	Imatinib 800 mg daily	Duplication at codons 502–503
*KIT* secondary mutations (exon 13, 14, 17, 18)	Acquired	Various (post-imatinib)	Resistant to imatinib/sunitinib; variable by exon	Sunitinib → regorafenib → ripretinib	Secondary resistance via ATP-binding/activation loop
*PDGFRA* exon 18 (D842V)	~5–8%	Gastric; epithelioid	Resistant to imatinib/sunitinib; sensitive to avapritinib	Avapritinib	D842V-specific resistance; test by NGS or PCR
*PDGFRA* non-D842V	< 2%	Gastric; variable	Sensitive to imatinib	Imatinib 400 mg	Includes exon 12/14; test by sequencing
*SDH*-deficient (*SDHx* mutation or methylation)	~5–7%	Gastric; multifocal; pediatric	Limited benefit from Imatinib; evidence evolving	Clinical trial; regorafenib in selected cases	*SDHB* IHC loss → methylation analysis
*NF1*-associated	Rare	Duodenum; small bowel	Limited benefit from Imatinib	Clinical trial; individualized management	*NF1* testing; usually lacks *KIT*/*PDGFRA* mutation
*BRAF* V600E	Very rare	Variable	Imatinib-resistant	*BRAF*/MEK inhibitors (off-label)	Test *BRAF* V600E via NGS
*NTRK* fusion	Very rare	Pediatric/*SDH*-wild-type	Sensitive to TRK inhibitors	Larotrectinib/Entrectinib	Test via IHC screening; confirm by fusion assay/NGS
FGFR fusion	Very rare	Variable	Potential target; evidence evolving	Clinical trial; FGFR inhibitor in selected cases	RNA fusion confirmation; broad NGS panel
Triple-negative/Wild-type	Rare	Variable; requires full profiling	Heterogeneous; profile-driven	Comprehensive profiling; clinical trial	Full NGS with CNV, fusion, and methylation panels

ATP: adenosine triphosphate; *BRAF*: B-Raf proto-oncogene; CNV: copy number variation; FGFR: fibroblast growth factor receptor; GIST: gastrointestinal stromal tumour; IHC: immunohistochemistry; *KIT*: *KIT* proto-oncogene receptor tyrosine kinase; MEK: mitogen-activated protein kinase kinase; N/A: not applicable; *NF1*: neurofibromin 1; NGS: next-generation sequencing; *NTRK*: neurotrophic tyrosine receptor kinase; PCR: polymerase chain reaction; *PDGFRA*: platelet-derived growth factor receptor alpha; *SDH*: succinate dehydrogenase; *SDHB*: succinate dehydrogenase subunit B; *SDHx*: succinate dehydrogenase complex subunit genes; TKI: tyrosine kinase inhibitor; TRK: tropomyosin receptor kinase.

**Figure 2:**
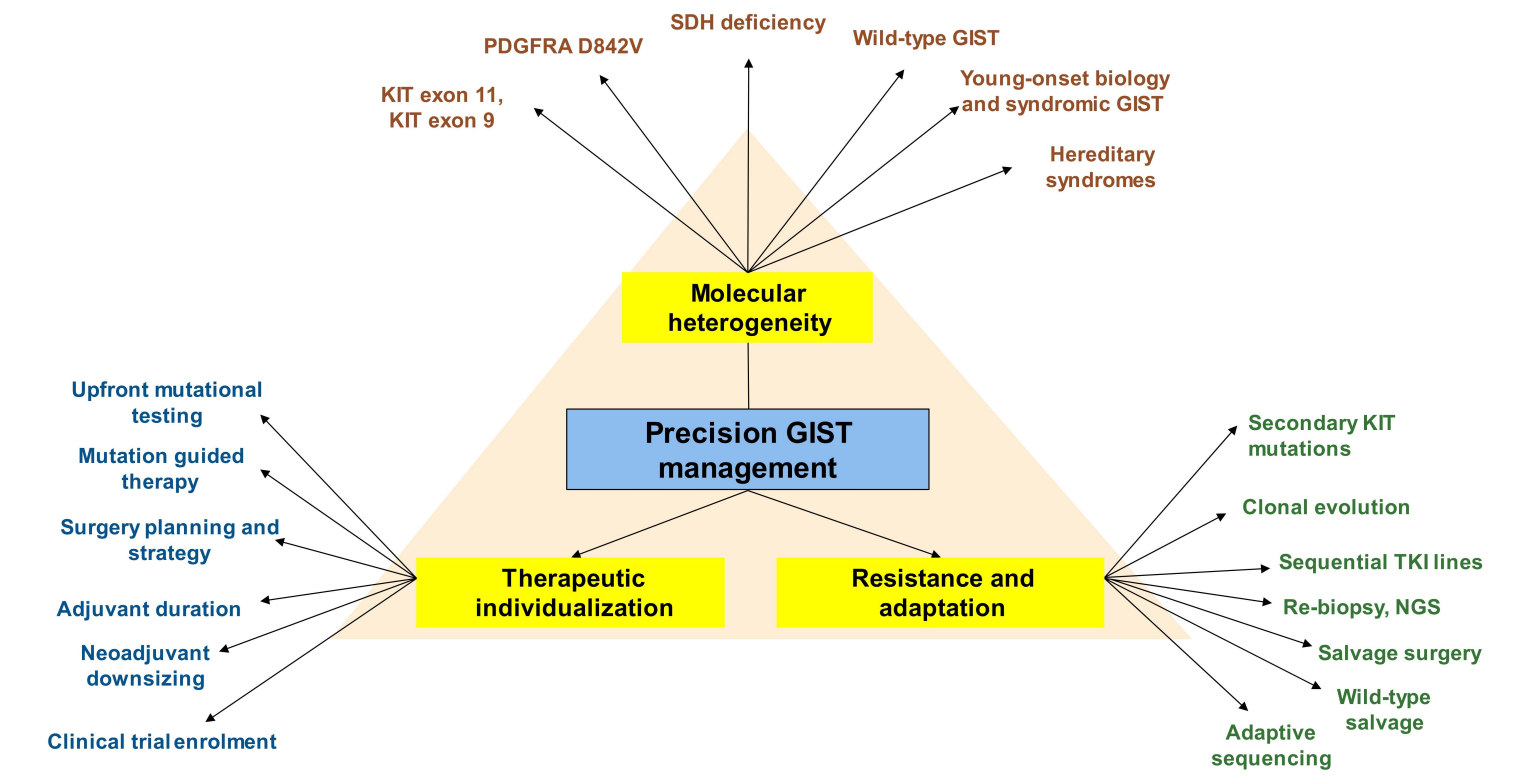
**Precision oncology framework for GIST.** GIST: gastrointestinal stromal tumour; *KIT*: *KIT* proto-oncogene receptor tyrosine kinase; NGS: next-generation sequencing; *PDGFRA*: platelet-derived growth factor receptor alpha; *SDH*: succinate dehydrogenase; TKI: tyrosine kinase inhibitor.

In addition to pharmacological management choices, it is essential to also consider organ system directed therapies for patients with metastatic disease. For example, a patient with liver metastases may be safely managed with liver ablation [39] or yttrium-90 radioembolization [40] following a multidisciplinary discussion. As survival extends with chronic TKI exposure, quality of life becomes a critical therapeutic endpoint. Patient-reported data show severe fatigue in 30% of patients with GIST, including 33% of those on TKIs, and severely fatigued patients have substantially worse global quality of life and impairment across physical, role, emotional, cognitive, and social functioning [41]. In selected patients with advanced GIST, surgery may retain a palliative or cytoreductive role as an adjunct to TKI therapy, particularly for symptom control, limited progression, or low-volume metastatic disease. In a meta-analysis of 9 studies including 1,416 patients, surgery combined with TKI therapy was associated with improved OS (HR 0.68, 95% CI 0.54–0.85) and PFS (HR 0.50, 95% CI 0.33–0.76) compared with TKI therapy alone [42]. However, in a multicentre EORTC-STBSG study of 239 patients undergoing metastasectomy in the imatinib era, incomplete R2 resection did not appear to prolong survival, suggesting that debulking surgery should be reserved mainly for symptom relief rather than expected oncologic gain [43]. Hence, a multidisciplinary team-directed individualized management plan is essential to ensure personalized medicine reaps survival gains at minimal cost to functional and quality-of-life outcomes. Figure 2 provides a summary of diverse considerations for personalized management of GIST patients.

## Tumours unresponsive to TKIs targeting *KIT*/*PDGFRA* alterations

Despite advances in TKI therapy, mutation-guided sequencing, and emerging targeted strategies, a subset of GISTs remains unresponsive to *KIT*/*PDGFRA*-directed treatment. These TKI-refractory tumours often exhibit limited responsiveness to conventional *KIT*/*PDGFRA*-directed TKIs, and effective systemic options remain poorly defined [44, 45]. Moreover, secondary resistance frequently arises through the acquisition of additional oncogenic alterations over the course of treatment, further complicating long-term disease control. A particularly relevant example is the growing interest in MEK-based strategies for *KIT*/*PDGFRA*-wild-type or molecularly complex GIST. The rationale is biologically strong: MAPK signalling stabilises ETV1, a master regulator of GIST lineage survival, and MEK inhibition alone may be insufficient because of feedback reactivation of upstream receptor tyrosine kinases such as *KIT* or *PDGFRA*. This has prompted interest in combined *KIT* and MEK blockade rather than MEK monotherapy. In a small phase I study of binimetinib plus pexidartinib in imatinib-refractory advanced GIST, treatment was well tolerated with no dose-limiting toxicities; the only grade 3 event was asymptomatic creatine phosphokinase elevation [46]. Although the study closed early after only two treated patients, one heavily pretreated patient achieved PFS of 6.1 months, while another patient with *NF1*-mutant GIST experienced a 27% RECIST tumour reduction and remained on therapy for 19 months, providing an important proof-of-concept that MEK-containing combinations may be particularly relevant in *NF1*-driven or *KIT*/*PDGFRA*-wild-type disease. These findings should be interpreted cautiously. The study enrolled only two treated patients and closed early; therefore, the results are best viewed as hypothesis-generating rather than evidence of established clinical benefit. Nevertheless, they provide biological support for further study of MEK-containing combinations, particularly in *NF1*-driven or *KIT*/*PDGFRA*-wild-type GIST. Single-cell data suggest that resistance is partly microenvironmental. In 9 specimens from 7 patients, scRNA-seq of 65,576 cells showed higher Treg infiltration and reduced cytotoxic CD8+ T-cell representation in imatinib-resistant GIST [47]. Resistant tumours showed TIGIT-NECTIN2 signalling involving Treg cells, and enrichment of IDO1-positive dendritic cells interacting through BTLA-TNFRSF14.

## Emerging directions in molecular stratification and resistance

Despite its success, precision oncology in GIST has important limitations. Genotype is highly informative but does not fully determine outcome, because treatment response is also shaped by tumour burden, anatomical site, rupture status, prior therapy, pharmacokinetics, patient tolerance, and clonal heterogeneity. Acquired resistance is often polyclonal, with secondary *KIT* or *PDGFRA* mutations emerging across different metastatic deposits, making a single tissue biopsy potentially incomplete. Liquid biopsy may improve detection of heterogeneous resistance, but circulating tumour DNA (ctDNA) sensitivity depends on tumour burden, disease biology, assay design, and timing of sampling. Rare subtypes such as *SDH*-deficient, *NF1*-associated, and other *KIT*/*PDGFRA*-wild-type GISTs remain therapeutically challenging because trial populations are small and drug sensitivity is less predictable. Therefore, molecular profiling should be viewed as a decision-support tool rather than a standalone treatment algorithm, and must be integrated with radiology, surgical feasibility, toxicity, quality of life, access to drugs, and multidisciplinary judgement.

Management of these challenging cases further highlights the importance of precision oncology to individualise patient therapies. This can involve salvage surgery with palliative intent, locoregional control strategies, and enrolment in clinical trials investigating next-generation inhibitors or immunomodulatory agents. This unmet therapeutic need underscores the urgency for translational research and innovative therapeutic approaches [48]. Recent multi-omic data extend GIST classification beyond single-driver genotyping. In a cohort of 117 GIST samples from 105 patients, GIST showed low tumour mutation burden but widespread copy number variation, and aggressive tumours harboured more genomic aberrations than low- or intermediate-risk lesions [49]. In the WES/WGS cohort, recurrent inactivating *YLPM1* mutations were identified in 7 of 68 patients (10.3%), and four molecular subtypes with distinct genomic, immune, and clinical features were proposed. Liquid biopsy may further refine this process. In the SCRUM-Japan pooled analysis, 44 patients underwent ctDNA analysis, and ctDNA was detectable in 72.7% [50]. ctDNA-positive patients had shorter PFS (HR 3.92), and subsequent mutation-matched TKI therapy was associated with longer PFS than unmatched treatment (8.23 vs. 2.43 months). Compared with tissue genotyping, blood genotyping identified more multi-exonic *KIT* alterations, including exon 11/13 (14.3% vs. 0%) and exon 13/17 (9.5% vs. 0%).

## Conclusions

Prior to the advent of TKIs, prognosis for patients with unresectable or metastatic disease was dismal, largely due to resistance to conventional cytotoxic chemotherapy and radiotherapy. The introduction of molecular-based therapies has since transformed the management paradigm and markedly improved clinical outcomes of GISTs. With the identification of *KIT* and *PDGFRA* mutations guiding first-line decisions, and emerging targets like *SDH*, *BRAF*, and *NTRK* informing novel strategies, the emphasis is shifting toward genotype-driven therapy. Both intrinsic and acquired resistance mechanisms underscore the importance of applying molecular surveillance to guide dynamic treatment sequencing. GIST management has evolved from a size- and site-based surgical paradigm to a genotype-informed therapeutic continuum in which molecular profiling determines not only systemic therapy selection but also the timing and intent of surgical intervention. As next-generation TKIs and biologically targeted agents continue to expand the therapeutic arsenal, multidisciplinary care, early mutational testing, and participation in clinical trials remain essential for optimising personalised GIST management and improving patient outcomes.

## Abbreviations

ACOSOG: American College of Surgeons Oncology Group
ACRIN: American College of Radiology Imaging Network
ATP: adenosine triphosphate
AYA: adolescent and young adult
*BRAF*: B-Raf proto-oncogene
ctDNA: circulating tumour DNA
DOG1: Discovered On GIST-1
EORTC: European Organisation for Research and Treatment of Cancer
ESMO: European Society for Medical Oncology
ETV1: Ets variant transcription factor-1
EUS: endoscopic ultrasonographic
FGFR1: fibroblast growth factor receptor 1
GI: gastrointestinal
GISTs: gastrointestinal stromal tumours
HR: hazard ratio
IGF1R: insulin-like growth factor 1 receptor
IHC: immunohistochemistry
*KIT*: *KIT* proto-oncogene receptor tyrosine kinase
MEK: mitogen-activated protein kinase kinase
NCCN: National Comprehensive Cancer Network
*NF1*: neurofibromin 1
NIH: National Institute of Health
*NTRK*: neurotrophic tyrosine receptor kinase
OS: overall survival
*PDGFRA*: platelet-derived growth factor receptor alpha
PFS: progression-free survival
PKC-theta: protein kinase C theta
R0: microscopically margin-negative resection
R1: microscopically margin-positive resection
RECIST: Response Evaluation Criteria in Solid Tumors
RFS: recurrence-free survival
RTOG: Radiation Therapy Oncology Group
*SDH*: succinate dehydrogenase
*SDHB*: succinate dehydrogenase subunit B
SEER: Surveillance, Epidemiology, and End Results
TKI: tyrosine kinase inhibitor
TRK: tropomyosin receptor kinase

## Declarations

### Copyright

© The Author(s) 2026.
